# Optimism and friendship quality as mediators between trait emotional intelligence and life satisfaction in Chinese adolescents: A two-wave longitudinal study

**DOI:** 10.1007/s12144-022-03931-0

**Published:** 2022-11-24

**Authors:** Xiaobo Wang, Xiong Lu, Tao Hu, Shuang Xue, Wenjian Xu, Wanjie Tang

**Affiliations:** 1 Department of Education and Psychology, Chengdu Normal University, Chengdu, China; 2 Faculty of Public Administration, Sichuan University, Chengdu, China; 3 Mental Health Centre, West China Hospital, Sichuan University, Chengdu, China; 4 Institute of Psychiatry, Psychology & Neuroscience, King’s College London, London, UK

**Keywords:** Emotional intelligence, Life satisfaction, Friendship quality, Adolescent, Optimism

## Abstract

Using a convenience sample of adolescents (*N* = 1609; 63.5% female; *M*_*age*_ = 16.54), this study explored whether EI predicted adolescent life satisfaction and whether friendship quality and optimism mediated this relationship during the COVID-19 pandemic. The structural equation modeling revealed that EI predicted adolescent life satisfaction, friendship quality, and optimism, friendship quality partially mediated the relationship between EI and life satisfaction, and optimism partially mediated the relationship between EI and friendship quality. These findings prove that psychological or educative approaches focused on EI could increase life satisfaction in adolescents during difficult times such as COVID-19, but EI may be linked with life satisfaction via friendship quality only. Training in optimism approaches and friendship quality enhancement programs could also effectively promote life satisfaction.

## Introduction

Since the COVID-19 outbreak in late 2019, millions of cases have been confirmed worldwide. The COVID-19 anti-infection strategies, such as social isolation, home confinement, and lockdowns, have resulted in significant fear and anxiety (Porcelli, 2020) and psychological health and well-being impairments (Sibley et al., 2020; Tang et al., 2020). COVID-19 has been a big challenge for teenagers as they have had to adapt to many new lifestyle changes, such as school closures, online home-based learning, and a reduction in interpersonal communication, all of which have had detrimental effects on adolescent mental health. Many studies have concluded that the COVID-19 pandemic has put adolescents at greater risk of developing psychological problems, stress, fear, and depressive symptoms (Hu et al., 2021; Jian et al., 2022; Magson et al., 2021; van der Laan et al., 2021). It has been suggested that life satisfaction could buffer the adverse effects of stressful life events and reduce the risk of psychological disorders (Suldo & Huebner, 2004). Previous studies have consistently shown that high satisfaction with life is a predictive factor for subjective well-being outcomes in adolescents (Castellá Sarriera et al., 2015). As promoting life satisfaction and preventing emotional and behavioral problems in children and adolescents have become important public policy goals during the COVID-19 pandemic, it is important to understand the factors and pathways that influence their life satisfaction.

### Literature review

Trait emotional intelligence (EI) is a constellation of emotion-related dispositions in which people identify, express, regulate and utilize their emotions and those of others to perceive their thinking and guide actions to adapt optimally (Mikolajczak, 2009; Petrides & Furnham, 2000; Petrides et al., 2007) (All abbreviations of EI hereafter in this article are referred to as trait EI). Trait EI is assessed through validated self-assessment questionnaires rather than through maximization tests (Austin, 2004). Extensive research has found a positive association between EI and life satisfaction, with higher EI being broadly related to higher life satisfaction (Gannon & Ranzijn, 2005; Kong et al., 2019), and an EI theoretical framework (Austin et al., 2005) considered that EI was related to high psychological well-being. Because people with high EI are better able to perceive, understand, use, and manage their feelings and those of others (Mayer & Salovey, 1997), they can effectively regulate themselves and others (Mayer & Salovey, 1995), which could contribute to well-being (Sánchez-Álvarez et al., 2016). Therefore, it could be surmised that good EI could assist in overcoming COVID-19-related stressors by increasing emotional and problem-focused coping skills, facilitating a greater sense of life satisfaction (Koçak, 2021). However, few studies have examined the internal mechanism between EI and life satisfaction in adolescents during the COVID-19 pandemic.

Dispositional optimism is a psychological construct that has been traditionally related to psychological well-being (Scheier & Carver, 1992). Optimism is defined as a generalized positive outcome expectancy, with optimists generally believing that positive rather than negative things are going to happen to them (Scheier & Carver, 1985). Theoretical analyses suggest that optimism is related to high life satisfaction because optimists tend to manage critical life situations more effectively (Scheier & Carver, 1993), tend to favor positive attributions, and are able to maintain a positive mental status more frequently and for longer, which could also enhance their sense of life satisfaction (Peterson & Seligman, 1984). A growing body of research has confirmed the positive relationship between optimism and life satisfaction (Bailey et al., 2007; Blasco-Belled et al., 2022; Martínez-Martí & Ruch, 2017; Oriol et al., 2020; Song et al., 2022). Therefore, optimism may be a crucial and positive predictor of life satisfaction in younger kids.

Several studies have also confirmed the relationship between EI and optimism (Kumcagiz et al., 2011; Tejada-Gallardo et al., 2020). In a cross-sectional study, a positive, robust association between optimism and EI was also found in younger students (Kumcagiz et al., 2011). However, there is little relevant adolescent data on this mediation mechanism, especially under COVID-19.

Friendship quality, which for adolescents is defined as the perceived support from friends (Furman & Buhrmester, 1992) might also play a key role in linking EI and well-being. A recent study found that the quality of friendship networks mediated the relationship between EI and subjective well-being in a college student sample (Zhang et al., 2020). High-quality friendships between adolescents are characterized by closeness, warmth, companionship, and positive reciprocity (Waldrip et al., 2008), with teenagers that had high-quality friendships being found to be better socio-emotionally adjusted and accepted by their peers (Flynn et al., 2017) and to have higher life satisfaction. Adolescents with higher quality friendships have also reported greater degrees of happiness, self-esteem, and life satisfaction (Raboteg-Saric & Sakic, 2014). Therefore, friendship quality may serve a vital role in EI and life satisfaction during the COVID-19 pandemic.

Empirical studies have also shown that optimism can improve the quality of adolescent friendships (Brissette et al., 2002). To date, there is limited knowledge on the mediating effects of friendship quality on the relationship between EI and life satisfaction under the COVID-19 pandemic and the role optimism plays in mediating the relationship between EI and friendship quality, particularly in adolescents.

### The current study

Using longitudinal mediation models, the current study sought to examine the effects of EI on later adolescent life satisfaction, the role optimism played as a mediator of these linkages, and the role of friendship quality in these processes during the COVID-19 pandemic. Based on previous studies and the previous discussion, it was hypothesized that higher levels of EI would be associated with later adolescent life satisfaction. Given the associations of optimism with EI and life satisfaction, it was also expected that optimism would mediate the relationships between EI and adolescent life satisfaction. In addition, considering relative stability of trait EI (Parker et al., 2005) and the relatively short time interval in this study, we considered EI to predict friendship quality rather than friendship quality to predict EI. Therefore, it was expected that friendship quality would mediate the relationships between EI and adolescent life satisfaction. Finally, as a robust predictor for positive outcomes, optimism was expected to mediate between EI and friendship quality. Structural equation modeling (SEM) was employed to explore the directions and relationships between these variables.

## Methods

### Study design and participants

The Survey of Behavior and Psychological Health Project on COVID-19 (SBPHP_ COVID-19) is an ongoing longitudinal study on the changes in people’s psychology during and after the COVID-19 pandemic. The study began collecting baseline information from February 24 to February 28, 2020, which was about one month after the nationwide lockdown in China (January 21), for which 2090 out of 2516 adolescents agreed to participate. A follow-up survey was then conducted with 1609 of the original 2090 adolescents from July 11 to July 23, 2020. These adolescent samples were students at two senior high schools in Leshan and Jianyang counties, Sichuan Province, China, who ranged in age from 12 to 18 (*M*_*age*_ = 16.54, *SD* = 0.98), with 63.5% being female. In the first school (Leshan), 718 students participated in the first wave, and 116 were lost to follow-up in the second wave. The second school (Jian Yang) had 1372 participants in the first wave and 364 failed to follow up in the second round. The descriptive statistical results are shown in Table 1. Because of the pandemic, Chinese students in these areas did not return to school (home quarantine) until April 1, 2020, and after about three months, summer vacation began on July 25.

**Table 1:** Demographic and exposure variables. (*N* = 1609)

Variables	*n*	%
Total	1609	100
Gender		
Male	588	36.54
Female	1021	63.46
Age (yr)		
≤ 15	243	15.10
16	526	32.69
17	552	34.31
18	288	17.90
Grade		
10	485	30.14
11	692	43.01
12	432	26.85
Only-child status		
Yes	397	24.67
No	1212	75.33
Type of exposure		
Felt extreme fear		
Yes	243	15.10
No	1366	84.90
Someone in the community infected		
Yes	92	5.72
No	1517	94.28
Family members infected		
Yes	1	< 0.01
No	1608	> 99.99
Relatives infected		
Yes	1	< 0.01
No	1608	> 99.99
Friends infected		
Yes	3	0.19
No	1606	99.81
Neighbors infected		
Yes	8	0.50
No	1601	99.50
Living in the worst-hit areas		
Yes	49	3.05
No	1560	96.95
Know someone who died of the infection		
Yes	3	0.19
No	1606	99.81

This study was conducted through the assistance provided by the Sichuan Psychological Society and helped establish relations with the local education department and two schools. Before the baseline survey, permission was obtained from the two school administrations and the local school board, after which the parents were informed of the purpose and significance of the assessment. The parents who agreed and their respective children then signed an electronic informed consent. An online questionnaire was then sent to parents and students via the WeChat App, and the student ID numbers and the last four digits of their parents' mobile phone numbers were taken to allow for tracking in this study. Ethics approval was granted by the Ethics Committee of the Sichuan University.

### Measures

In the baseline assessments (T1), the participants were required to provide information about their gender, age, grade, COVID-19 pandemic exposure, and EI. The second wave (T2) was conducted about six months later, collecting information on the participants’ friendship quality, optimism, and life satisfaction between T1 and T2.

#### Covariates

Informed by the life satisfaction literature (van der Laan et al., 2021), gender and COVID-19 pandemic exposure were included as covariates in this study. COVID-19 pandemic exposure was measured using an eight-item scale from a modified version of the disaster exposure scale evaluating objective and subjective features of exposure: Has anyone in your community or village been infected? Has anyone in your family been infected? Have any of your friends or classmates been infected? Have any of your relatives been infected? Has anyone in your neighborhood been infected? Are you extremely afraid of the COVID-19 pandemic? Is there a severe COVID-19 outbreak in your area? Has anyone in your vicinity (including your relatives, friends, and classmates) died of a COVID-19 infection? Respondents answered these items with a “yes” or “a no”. The Cronbach’s α for the scale was 0.65 in the current study. In the present study, the total score was calculated by adding up the yes responses.

#### EI

EI was assessed using the Wong Law Emotional Intelligence Scale (WLEIS) (Wong & Law, 2002), which has been found to have good reliability and validity in a Chinese sample (Li et al., 2021). This scale has sixteen items that assess the four factors related to EI; emotional regulation, self-emotion appraisal, emotion appraisal of others, and the use of emotion; to which participants responded on a Likert-type scale ranging from 1 (never) to 5 (always), with higher scores indicating higher EI. The Cronbach’s α for the scale was 0.97 in the current study.

#### Life satisfaction

Life satisfaction was assessed using the Satisfaction with Life Scale (SWLS) (Diener et al., 1985), which has been found to have good levels of reliability and validity in Chinese adolescents (Lu et al., 2020). Participants responded to five items, such as “During the last five months, I am satisfied with my life”, on a 7-point Likert scale from 1 (strongly disagree) to 7 (strongly agree), with higher scores reflecting higher life satisfaction (Lu et al., 2020). The Cronbach’s α for the present sample was 0.92.

#### Friendship quality

Adolescent friendship quality was assessed using the Friendship Quality Scale (Valkenburg & Peter, 2007), which has also been found to have satisfactory reliability and validity in Chinese youth (Wang et al., 2014). The scale has four items, such as “During the last five months, when my friends know that something is bothering me, they ask me about it”, which was rated on a 5-point Likert scale from 1 (strongly disagree) to 5 (strongly agree), with higher scores reflecting higher friendship quality (Wang et al., 2014). The Cronbach’s α in the present study was 0.94.

#### Optimism

Optimism during the last five months was measured using the six-item Life Orientation Test scale (LOT) (Scheier & Carver, 1985), which participants responded to using a five-point Likert type scale ranging from 1 (strongly disagree) to 5 (strongly agree), with higher scores reflecting higher optimism. The LOT has been widely used on Chinese adolescent samples and has been found to have good reliability and validity (Lai & Yue, 2000). The Cronbach’s α for the scale was 0.78 in the current sample.

#### Data analysis

SPSS software 22, the arithmetical program Process 3.0 (Hayes, 2017), and AMOS software were used to process the data. Descriptive statistics were used to calculate the subjects' demographic and pandemic-related variables, Pearson’s correlation was used to calculate the relationships between the main continuous variables, Model 4 mediation analysis was used to analyze the mediating effects between each variable, and SEM was used to analyze the paths and relationships between the four main variables. Because of the lack of a standard format for reporting fit, to examine whether the hypothesized model fits the observed data, four recommended indices were applied: (a) the Root Mean Square Error of Approximation (RMSEA) with a good fit being when the RMSEA is less than 0.08; (b) the comparative fit index (CFI); (c) the Tucker-Lewis index (TLI); and (d) the Goodness of Fit Index (GFI), with a good fit being when the TLI, GFI, and CFI values were greater than 0.90. the chi-square test significance of this model was 0.05 or less.

## Results

### Preliminary results

The means, standard deviations, and correlations between the variables are summarized in Table 2. There was no significant difference in trait EI between participants who completed the second survey and those who did not complete the follow-up survey(72.83 ± 20.26 vs. 71.58 ± 17.80, *t* = 1.22, *p* = 0.22). Most variables were normally distributed with no obvious skewedness (-1.08 to -0.05), although the kurtosis for the optimism values (2.23) fell slightly above the recommended values of plus or minus two. No multicollinearity was found in the preliminary bivariate correlation analyses, indicating that the independent variables were not highly correlated. EI was found to have moderate, positive associations with optimism, friendship quality, and life satisfaction.

**Table 2:** Descriptive statistics and correlation coefficients (*N* = 1609)

Variables	1	2	3	4	5	6	7
1. Gender	1						
2. Age	-0.06^*^	1					
3. COVID-19 pandemic exposure	0.08^**^	-0.01	1				
4. Emotional Intelligence	-0.08^**^	-0.03	0.08^**^	1			
5. Optimism	-0.02	-0.01	0.02	0.48^**^	1		
6. Friendship Quality	-0.09^**^	-0.05	0.01	0.55^**^	0.37^**^	1	
7. Life Satisfaction	-0.07^**^	-0.01	-0.01	0.50^**^	0.34^**^	0.52^**^	1
Range	1–2	12–18	15–18	16–112	6–30	4–20	5–33
Mean	1.63	16.54	17.50	72.83	18.99	14.09	19.70
SD	0.48	0.98	0.64	20.26	4.26	3.57	6.80
Skewness	-0.56	-0.23	-1.08	-0.90	-0.60	-1.01	-0.05
Kurtosis	-1.69	-0.26	0.68	1.23	2.23	1.22	0.05

^*^p < 0.05, ^**^p < 0.01

### Testing for the mediation models

A series of mediation models were used to assess the potential individual mediating effects of optimism and friendship quality. As shown in Table 3, after controlling for gender and COVID-19 pandemic exposure, the adolescent reported optimism was found to mediate the effects of EI on adolescent life satisfaction over time. Based on 5000 bootstraps resamples, the indirect path from EI to optimism to adolescent life satisfaction was significant (indirect effect = 0.02, 95% CI: [0.01, 0.03]), with a reduction of 12% in the total effect. Similarly, the adolescent reported friendship quality also partially mediated the effects of EI on adolescent life satisfaction over time. The indirect path from EI to friendship quality to adolescent life satisfaction was significant (indirect effect = 0.07, 95% CI: [0.05, 0.08]), with a reduction of 39% in the total effect. The adolescent reported optimism also mediated the effects of EI on adolescent friendship quality (indirect effect = 0.02, 95% CI: [0.01, 0.02]), with reductions of more than 12% in the total effects.

**Table 3:** Mediation analyses to assess the direct and indirect effects between emotional intelligence and life satisfaction through optimism and friendship quality (*N* = 1609)

Path	Effect	*SE*	*p*	95% CI
Emotional intelligence (X) → Optimism (M) → Life satisfaction (Y)				
The total effect of X on Y	0.17	0.007	< 0.001	[0.15, 0.18]
The direct effect of X on Y	0.15	0.008	< 0.001	[0.13, 0.16]
The indirect effect of X on Y	0.02	0.006	< 0.001	[0.01, 0.03]
X → M	0.10	0.005	< 0.001	[0.09, 0.11]
M → Y	0.21	0.039	< 0.001	[0.13, 0.28]
Emotional intelligence (X) → Friendship quality (M) → Life satisfaction(Y)				
The total effect of X on Y	0.17	0.007	< 0.001	[0.15, 0.18]
The direct effect of X on Y	0.10	0.008	< 0.001	[0.08, 0.12]
The indirect effect of X on Y	0.07	0.007	< 0.001	[0.05, 0.08]
X → M	0.10	0.004	< 0.001	[0.09, 0.10]
M → Y	0.68	0.046	< 0.001	[0.59, 0.77]
Emotional intelligence (X) → Optimism (M) → Friendship quality(Y)				
The total effect of X on Y	0.10	0.004	< 0.001	[0.09, 0.10]
The direct effect of X on Y	0.08	0.004	< 0.001	[0.08, 0.09]
The indirect effect of X on Y	0.02	0.003	< 0.001	[0.01, 0.02]
X → M	0.10	0.005	< 0.001	[0.09, 0.11]
M → Y	0.12	0.020	< 0.001	[0.08, 0.16]

^a^ Bootstrap SEs and 95% CIs were calculated in the indirect effect

Abbreviations: SE, standard error; CI, confidence interval

### Structural equation models

**Fig. 1:**
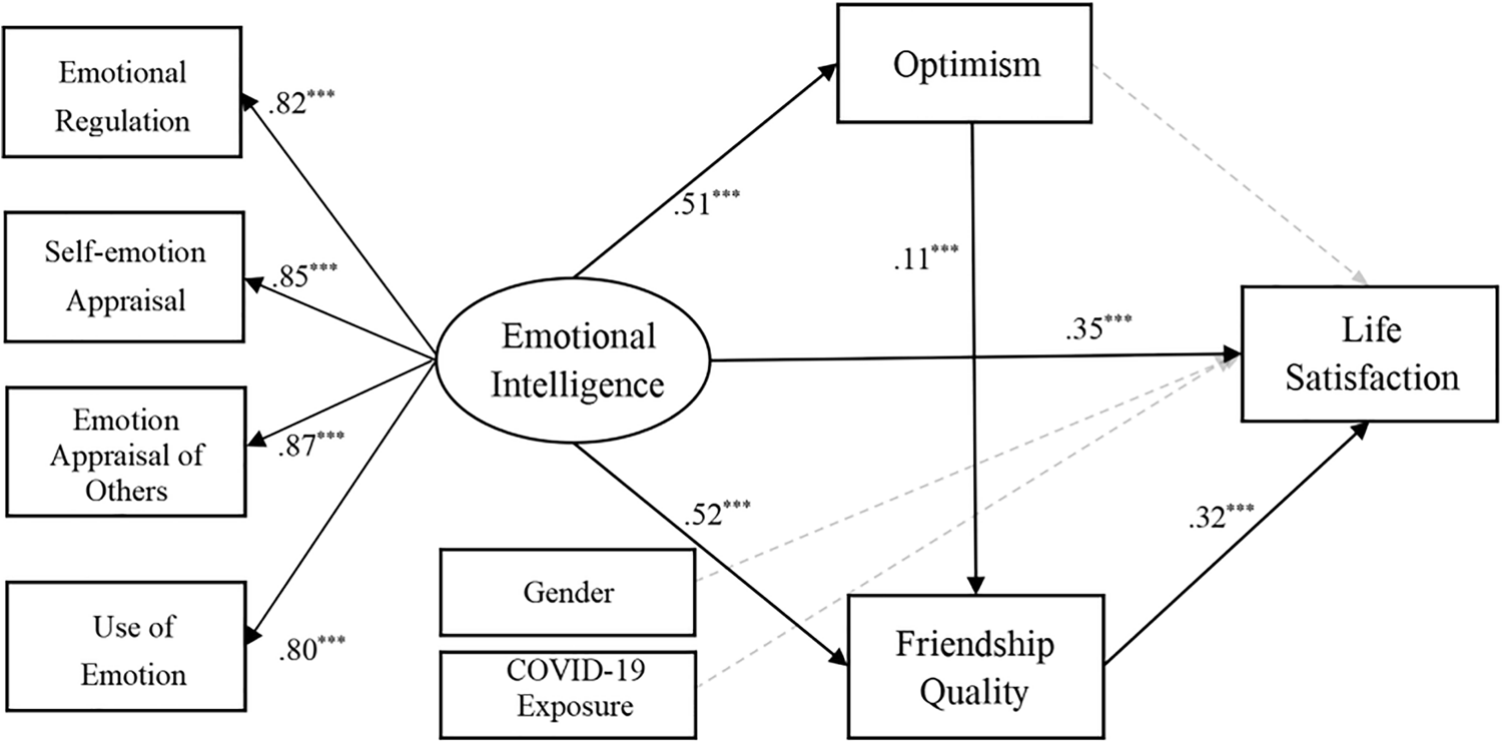
Structural Equation Model with standardized coefficients from T1 emotional intelligence to T2 positive consequences in the adolescent responses during the COVID-19 pandemic. Notes: ^***^p < 0.001

The hypothetical structural equation model was tested. The invisible pathways from the covariates to the key variable were included and tested in the final model. The standardized coefficients were adjusted for the effects of adolescent gender and COVID-19 pandemic exposure. As shown in Fig. 1, the final mediation model had a chi-square of 186.6 (*p* < 0.001) with 25 degrees of freedom, an RMESA of 0.06, a CFI of 0.97, a GFI of 0.98, and a TLI of 0.96, all of which indicated a good fit to the data. The structural parameters showed a significant positive pathway from EI to life satisfaction (*β* = 0.35, *p* < 0.001), optimism (*β* = 0.51, *p* < 0.001), and friendship quality (*β* = 0.52, *p* < 0.001), which suggested that adolescents with high EI were more likely to perceive positive outcomes. Further, a significant indirect effect of friendship quality was found for the association between EI and life satisfaction.

## Discussion

The present study explored the pathways for positive psychological characteristics in adolescents who had experienced a period of national lockdown in China due to the COVID-19 pandemic and had gone through a readjustment stage after returning to school. The SEM analysis revealed that EI predicted adolescent optimism, friendship quality, and life satisfaction, friendship quality partially mediated the relationship between EI and life satisfaction, and optimism partially mediated the relationship between EI and friendship quality. These findings highlighted the important role played by EI and friendship quality in adolescent life satisfaction and the underlying mechanisms in these relationships. Therefore, developing adaptive training programs that enhance emotional competencies for processing, facilitating, understanding, and managing emotions could be useful in promoting higher adolescent life satisfaction.

These results indicated that EI predicted life satisfaction among adolescents, which accords with the findings of previous research on the association between EI and life satisfaction in adolescents (Blasco-Belled et al., 2020; Prado Gascó et al., 2018; Ramos-Díaz et al., 2019; Villanueva et al., 2022) and young adults (Kong & Zhao, 2013; Koydemir et al., 2013; Liu et al., 2013; Zhao et al., 2021). A previous meta-analysis found that EI was highly correlated with life satisfaction (Sánchez-Álvarez et al., 2016). Therefore, the findings in this study extend previous COVID-19 pandemic research by highlighting the essential role played by EI in life satisfaction using a prospective design (Koçak, 2021; Tariq et al., 2021). The possible explanation for this association is that as a set of mental abilities, EI involves the ability to perceive, understand, express, and regulate one’s own emotions and to discriminate feelings (Schutte et al., 2001), all of which lead to more positive emotional states, reduced negative feelings (Kong et al., 2019), and a greater sense of health and well-being (Sánchez-Álvarez et al., 2016). As EI often refers to the trait of emotional self-efficacy, which manifests itself in specific attributes, such as empathy and assertiveness (Petrides & Furnham, 2000), and life satisfaction is an overall cognitive evaluation of life (Lewinsohn et al., 1991); it appears that friendship quality could act as a bridge and mediator between these two positive psychological variables. Therefore, teenagers with high EI would be able to better apply this emotional competence to gain more friendships and greater value and happiness, which would then result in a more positive overall life satisfaction.

To the best of our knowledge, this is the first study that has identified the mediating friendship quality mechanism that underlies the relationship between EI and life satisfaction in adolescents. This research suggested that EI predicts friendship quality over time, which in turn promotes life satisfaction. This result was partly consistent with a previous cross-sectional study that found that the central position of friendship networks mediated the relationship between EI and subjective well-being in a small sample of university students (Zhang et al., 2020). Adolescents usually spend a lot of time with their friends as close friendships provide them with developmentally salient opportunities to improve their social skills and social competencies (Collins & Steinberg, 2007). High friendship quality also leads to high self-esteem, better peer acceptance (Keefe & Berndt, 1996), and better support (Bakalım & Taşdelen-Karçkay, 2016), which generally leads to better social adaptation and social adjustment abilities (Hartup & Stevens, 1997) and significant increases in life satisfaction over time (Demir & Özdemir, 2010). A high-quality friendship is characterized by high levels of prosocial behavior, intimacy, self-esteem support, and loyalty (Berndt, 2002), which not only directly promotes life satisfaction in teenagers but also stimulates the EI mechanism mediating life satisfaction.

The SEM in this research also identified another path where EI promoted optimism, which in turn improved friendship quality. These results extended previous research that found that EI was positively associated with optimism (Kumcagiz et al., 2011). EI was found to contribute to the development of the traits associated with a positive outlook and confidence in the future; however, these results need to be confirmed over a longer period. It has been previously proposed that optimists have adequate and larger social support networks (Brissette et al., 2002), which could partly explain the higher friendship quality for the optimists in our data. These results could assist in understanding the developmental mechanisms and paths for positive adolescent psychological development and provide a reference for future education interventions.

### Theoretical and practical implications

The findings in this study have important theoretical and practical implications. Although there is considerable empirical and theoretical support (Kong et al., 2019; Ruiz‐Aranda et al., 2014) for the effect of EI on adolescent positive outcomes, this study was the first to reveal the long-term impacts of EI on adolescent life satisfaction and the underlying mechanisms. In particular, this study provided preliminary evidence for how EI affects positive adolescent development through friendship quality and also identified optimism as a distinctive mediation link to adolescent EI and friendship quality.

The results of the study echo findings from previous trait EI research in both adults and adolescents, highlighting the positive role of enhancing mental well-being. For example, a comprehensive review showed that EI training improved individual well-being, life satisfaction, and quality of life and even significantly affected adolescent academic achievement (Petrides et al., 2016). Another special issue on trait EI by Pérez-González et al. emphatically emphasized the usefulness of considering trait EI as a key explanatory variable in personal, social, educational, and vocational development at a theoretical level (Pérez-González et al., 2020).

These findings also provide valuable guidance on psychological interventions that can enhance adolescent life satisfaction. As researchers have argued that EI can be improved through training programs or experience (Geßler et al., 2021), interventions to improve adolescent emotional regulation skills could be effective in alleviating the detrimental consequences of stressful events such as the COVID-19 pandemic and improving teenage life satisfaction. By understanding the dispositional predictors of life satisfaction, schools could design intervention programs to develop and improve EI, adolescent optimism, and interpersonal skills. Therefore, our results validate the conclusions of previous studies and expand the application context, even during the pandemic, trait EI could be a useful target, and improvement through EI training may help adolescents improve friendship quality, optimism, and life satisfaction. This also gives some implications to the education departments to consider establishing courses related to trait EI to help improve students' positive psychological feelings, well-being, and quality of life.

### Limitations and future studies

Regardless of the positive findings, some limitations should be considered when interpreting the results. First, as the use of self-assessment questionnaires and online assessments could have some effect on the reliability and validity of the assessments. For instance, it is widely observed that self-report attitudes or abilities generally correlate positively with one another (Podsakoff et al., 2012). Therefore, objective measurements and indicators could be considered in future studies to prevent this potential method bias. Second, a convenience sample from only two schools was used, which may have affected the representativeness; therefore, future studies should draw from a larger and more random sample. Third, as trait EI is widely observed to correlate strongly with more general personality traits (Gannon & Ranzijn, 2005; van der Linden et al., 2017, 2018), the potential influence of personality factors should be ruled out (Podsakoff et al., 2012). Thus, it would be a good direction for future research to see if our results can be replicated when ratings of EI and/or friendship quality are gathered from peers instead of all self-reported. Fourth, it could be the case that greater friendship support helps foster greater self-perceptions of EI, so future research is necessary to explore the predictive relationship between them. Lastly, although measured at different points in time, both the independent, mediating and dependent variables in this study were measured only once, which limited the ability to unravel the developmental changes in adolescents’ positive outcomes over time. Future studies need to use a cross-lagged panel design to analyze longitudinal data measured at two or more time points to fully delineate the causal relationship between EI and life satisfaction.

## Conclusions

Despite these limitations, this study advances knowledge on the relationships between EI and life satisfaction. As the EI was measured during the worst phase of the epidemic only one month after the national lockdown in China, and life satisfaction was measured at the end of the semester after students had returned to school, a distinct mechanism was found between these two positive psychological variables as it was found that EI predicted life satisfaction and also influenced life satisfaction by strengthening friendship quality. These findings are important because as the pandemic continues to affect lives around the world, they provide possible directions and paths to increase adolescent well-being and life satisfaction. Longer-term perspective studies on EI and life satisfaction could contribute to a better overall understanding of positive psychological traits and quality of life outcomes in adolescents facing major stressful life events.

## Data Availability

Data is available upon reasonable request.
